# Loss of *TNFAIP3* enhances *MYD88*_*L265P*_-driven signaling in non-Hodgkin lymphoma

**DOI:** 10.1038/s41408-018-0130-3

**Published:** 2018-10-09

**Authors:** Kerstin Wenzl, Michelle K. Manske, Vivekananda Sarangi, Yan W. Asmann, Patricia T. Greipp, Hanna R. Schoon, Esteban Braggio, Matthew J. Maurer, Andrew L. Feldman, Thomas E. Witzig, Susan L. Slager, Stephen M. Ansell, James R. Cerhan, Anne J. Novak

**Affiliations:** 1 Division of Hematology, Mayo Clinic, Rochester, MN USA; 2 Department of Health Sciences Research, Mayo Clinic, Rochester, MN USA; 3 Department of Health Sciences Research, Mayo Clinic, Jacksonville, FL USA; 4 Genomics Laboratory, Division of Laboratory Genetics and Genomics, Department of Laboratory Medicine and Pathology, Mayo Clinic, Rochester, MN USA; 5 Division of Hematology, Mayo Clinic, Phoenix, AZ USA; 6 Department of Laboratory Medicine and Pathology, Mayo Clinic, Rochester, MN USA

## Abstract

*MYD88* mutations are one of the most recurrent mutations in hematologic malignancies. However, recent mouse models suggest that *MYD88*_*L265P*_ alone may not be sufficient to induce tumor formation. Interplay between *MYD88*_*L265P*_ and other genetic events is further supported by the fact that *TNFAIP3* (A20) inactivation often accompanies *MYD88*_*L265P*_. However, we are still lacking information about the consequence of *MYD88*_*L265P*_ in combination with *TNFAIP3* loss in human B cell lymphoma. Review of our genetic data on diffuse large B cell lymphoma (DLBCL) and Waldenstrom macroglobulinemia (WM), found that a large percentage of DLBCL and WM cases that have a *MYD88* mutation also harbor a *TNFAIP3* loss, 55% DLBCL and 28% of WM, respectively. To mimic this combination of genetic events, we used genomic editing technology to knock out *TNFAIP3* in *MYD88*_*L265P*_ non-Hodgkin’s lymphoma (NHL) cell lines. Loss of A20 expression resulted in increased NF-κB and p38 activity leading to upregulation of the NF-κB target genes *BCL2* and *MYC*. Furthermore, we detected the increased production of IL-6 and CXCL10 which led to an upregulation of the JAK/STAT pathway. Overall, these results suggest that *MYD88*_*L265P*_ signaling can be enhanced by a second genetic alteration in *TNFAIP3* and highlights a potential opportunity for therapeutic targeting.

## Introduction

Next-generation sequencing data has revealed that *MYD88* mutations are one of the most recurrent mutations in hematologic malignancies and are found in 20% of lymphomas (COSMIC data base^1^). While it is detected in many subtypes of B cell malignancies, its prevalence is highest in Waldenstrom macroglobulinemia (90–100%, WM), primary CNS lymphomas (79%), and the activated B cell subtype of diffuse large B cell lymphoma (39%, ABC-DLBCL)^2–4^. The most common *MYD88* mutation described thus far is a single base pair mismatch resulting in an amino acid switch from lysine to proline at position 265 (*MYD88*_*L265P*_). MYD88 is an adaptor protein which acts downstream of the Toll-like receptor (TLR) and interleukin-1 pathways^5^. MYD88 activation leads to IRAK1/4 recruitment and further downstream activation and phosphorylation of TRAF6 and TAK1 resulting in NF-κB activation^6^. MYD88_L265P_ is a constitutively active form of the protein and its expression leads to dysregulated NF-κB and STAT3 signaling^2^. Additionally, we have shown in a recent study that TAK1 is an essential player in the MYD88_L265P_ pathway and that it contributes to cell proliferation and cytokine secretion^7^. Together, these data suggest that mutant forms of *MYD88* drive development of lymphoma. However, it has recently been shown in mouse models that *MYD88*_*L265P*_ alone is not sufficient to induce tumor formation and requires additional genetic hits, such as loss of the *TNFAIP3* tumor suppressor (encodes for the A20 protein) or *BCL2* upregulation^8,9^.

Deletion or mutations in *TNFAIP3* on 6q23 are commonly found in DLBCL and WM^10,11^, and when combined with a *MYD88* mutation may further lead to deregulated NF-κB activation. A20 is an inducible ubiquitin-modifying enzyme and part of the NF-κB-induced negative feedback loop^12^. Its role as a tumor suppressor gene in hematological malignancies has been shown in various studies, where restoring of A20 expression in A20 deficient cell lines lead to induction of apoptosis, cell growth arrest, and downregulation of NF-κB target genes^10,13,14^. Furthermore, it has been shown that A20 expression is rapidly induced in cells to counteract MYD88-driven proliferation and NF-κB activation^8^.

The mechanistic interplay and downstream consequence of *MYD88*_*L265P*_ in combination with additional genetic hits have not been fully defined in human lymphoma models of *MYD88*_*L265P*_. From a clinical perspective, further insight on *MYD88*-driven proliferation is important for therapeutic targeting of this pathway. TLR signaling inhibitors have been shown to have an effect on tumor growth in *MYD88* mutant cell line models and patient-derived DLBCL tumor xenograft mouse models^15,16^. Additionally, in a recent phase I/II clinical trial in relapsed or refractory ABC-DLBCL it was shown that 80% of patients who harbor a *MYD88* together with a *CD79B* mutation were sensitive to the B cell receptor (BCR) signaling inhibitor ibrutinib. The same study also showed that inactivation of *TNFAIP3* reduced ibrutinib response^17^. Novel therapeutic agents continue to be developed to target the MYD88_L265P_ pathway in both DLBCL and WM and delineation of the mechanism of how this mutation impacts tumor cells alone, or in combination with additional genetic hits, is of clinical significance. Therefore, the aim of this study is to investigate the cellular consequences of *MYD88*_*L265P*_ in combination with *TNFAIP3* inactivation in WM and DLBCL. Our studies demonstrate that co-occurrence of both genetic events has a significant impact on activation of NF-κB and p38. Additionally, we show that loss of A20 leads to elevated secretion of IL-6 and CXCL10, which further drives the activation of JAK/STAT3 pathway. Identification of patients who harbor both of these genetic variants may lead to the development of a genetic biomarker for individualized therapy.

## Material and methods

### Patients, whole-exome sequencing, and copy number analysis

This study was reviewed and approved by the human subjects review board of Mayo Clinic and the University of Iowa, and written informed consent was obtained from all participants. For DLBCL, identification of *MYD88* mutant cases was done using whole exome sequencing (WES) data from 145 newly diagnosed DLBCL tumors. WES data from tumor-normal pairs (*n* = 56)^7,18^ and tumors embedded in FFPE (*n* = 89) were combined and analyzed together as described in Supplemental Methods. *TNFAIP3* copy number loss was identified using WES (*n* = 56) or whole genome copy number data (*n* = 89) from the OncoScan array (Affymetrix, Santa Clara, CA, USA) and analyzed as described in Supplemental Methods. The cell of origin was determined using the Hans algorithm, gene expression profiling, or NanoString technology^19–21^. For WM, identification of *MYD88* mutant cases has been described previously using WES or allele-specific PCR (ASO-PCR)^7^ and *TNFAIP3* copy number loss was assessed using real-time quantitative PCR. Briefly, genomic DNA was extracted from 29 WM patients and a TaqMan™ copy number assays probe (Thermo Scientific, Waltham, MA) for *TNFAIP3* were used. All qPCR reactions were performed using BioRad CXF96 instrument and the results are expressed as relative units based on calculation 2^−ΔΔCT^, which gives the relative amount of target gene normalized to the endogenous control. A copy number loss was defined using a cutoff based the mean of the normal controls (*n* = 5) minus 4 standard deviations, a value of 0.8 was defined as the cutoff for *TNFAIP3* loss (Supplemental Figure 1).

### Cell lines and establishment of *TNFAIP3* knockout clones

The MWCL cell line was established and characterized at Mayo Clinic^22^. The BCWM cell line was a kind gift from Dr. Steve Treon and the HBL-1 cell line was kindly provided by Dr. Thomas Witzig. Cell line authentication is described in Supplemental Methods. All cells lines were maintained in RPMI 1640 medium with 10–20% fetal bovine serum (FBS), penicillin (50 U/ml), and streptomycin (50 µg/ml) were added. Cells were periodically checked for mycoplasma by PCR and were found to be negative. All cell lines were cultivated at 37 °C and 5% CO_2_. Transcription activator-like effector nuclease (TALENs) specific for targeting exon 5 of the *TNFAIP3* gene were designed by the Mayo Clinic Genetics and Model Systems Core using the FusX system as previously described^23^. Exon 5 was targeted because it is present in all reported *TNFAIP3* isoforms. Cells were transfected with 10 μg of each TALEN vector arm by using the AMAXA® nucleofection system (Amaxa, Cologne, Germany). To track successful transfection, cells were co-transfected with a 2 μg GFP expressing plasmid. After 48 h, cells that have incorporated the GFP expression plasmids were isolated by single cell sorting at the Mayo Flow Cytometry Facility. Restriction enzyme digest was used to initially identify clones that carried the frameshift mutation.

### Quantitative real-time PCR and RNA-Seq

Total RNA was extracted using the RNeasy Mini Plus Kit (Qiagen GmbH, Hilden, Germany) according to the manufacturer’s protocol. cDNA was synthesized using the SuperScript III First-Strand Synthesis kit (Invitrogen, Waltham, MA, USA). Quantitative reverse transcriptase-PCR was performed using either TaqMan probes (Applied Biosystems, Invitrogen, Carlsbad, CA) or probe-based predesigned qPCR primers (IDT, Coralville, IA, USA). All qPCR reactions were performed using BioRad CXF96 instrument. The results are expressed as relative units based on calculation 2^−ΔΔCT^, which gives the relative amount of target gene normalized to the endogenous control. For RNAseq, total RNA was extracted using the miRNeasy Mini Plus Kit (Qiagen GmbH, Hilden, Germany). Library preparation and RNA-sequencing were carried out by the Mayo Clinic Genome Analysis Core. Library preparation was done using the Standard TruSeq v2 for mRNA (Illumina, San Diego, CA, USA) and sequencing was carried out on Illumina HiSeq 4000 (Illumina, San Diego, CA, USA). Data analysis was performed by the Mayo Clinic Bioinformatics Core using the Mayo Clinic mRNA-Seq analysis pipeline MAPRSeq (v2.1.1.). A detailed description of the secondary analysis can be found in Supplemental Methods.

### Western blot analysis

Cells were lysed in RIPA buffer (Thermo Scientific, Waltham, MA, USA) with protease and phosphatase inhibitor cocktail (Thermo Scientific, Waltham, MA). Protein extracts were clarified by centrifugation, resolved by SDS-PAGE using PROTEAN® TGX™ gels (Bio-Rad Laboratories, Hercules, CA), and transferred to PVDF membranes. Antibodies used in the study are listed in Supplemental Methods.

### Cytokine expression analysis

Cells where cultured for 48 h under normal culturing conditions and samples were analyzed using the Human ProcartaPlex™ Simplex Kit for IL-6 and CXCL10 (Invitrogen, Waltham, MA, USA). Samples were run in duplicate and the assay was performed according the manufacturer’s instructions. Plates were read on a Luminex-200 system (Luminex, Austin, TX, USA) and analyzed using Star Station software (Applied Cytometry, Sheffield, UK).

### Proliferation assay

Proliferation assay methods have been described previously^7^. Briefly, cell lines were plated (10 or 25 × 10^3^ cells/well) in culture media in the presence of either DMSO (Thermo Scientific, Waltham, MA, USA) or ibrutinib (Chemitek, Indianapolis, IN, USA) at the indicated doses for 48 h. After 20 h of incubation, cells were pulsed with 0.05 mCi tritiated thymidine (Amersham, Piscataway, NJ, USA) and ^3^H-thymidine incorporation levels were determined using a MicroBeta TriLux (PerkinElmer, Waltham, MA, USA). All raw counts were normalized to the DMSO control by setting DMSO control to 100.

### Statistics

Statistical analyses were performed using GraphPad Prism 7.0 (GraphPad Software, USA). *p*-Values for all experiments have either been calculated using the 2-tailed Student *t*-test, or if applicable, the Wilcoxon–Mann–Whitney test was used. Fisher’s exact test was used to compare the correlation between genomic events. *p*-Value of ≤0.05 was considered to be statistically significant. All error bars are shown as standard deviation (SD).

## Results

### *MYD88* and *TNFAIP3* genetic alterations in DLBCL and WM

**Fig. 1:**
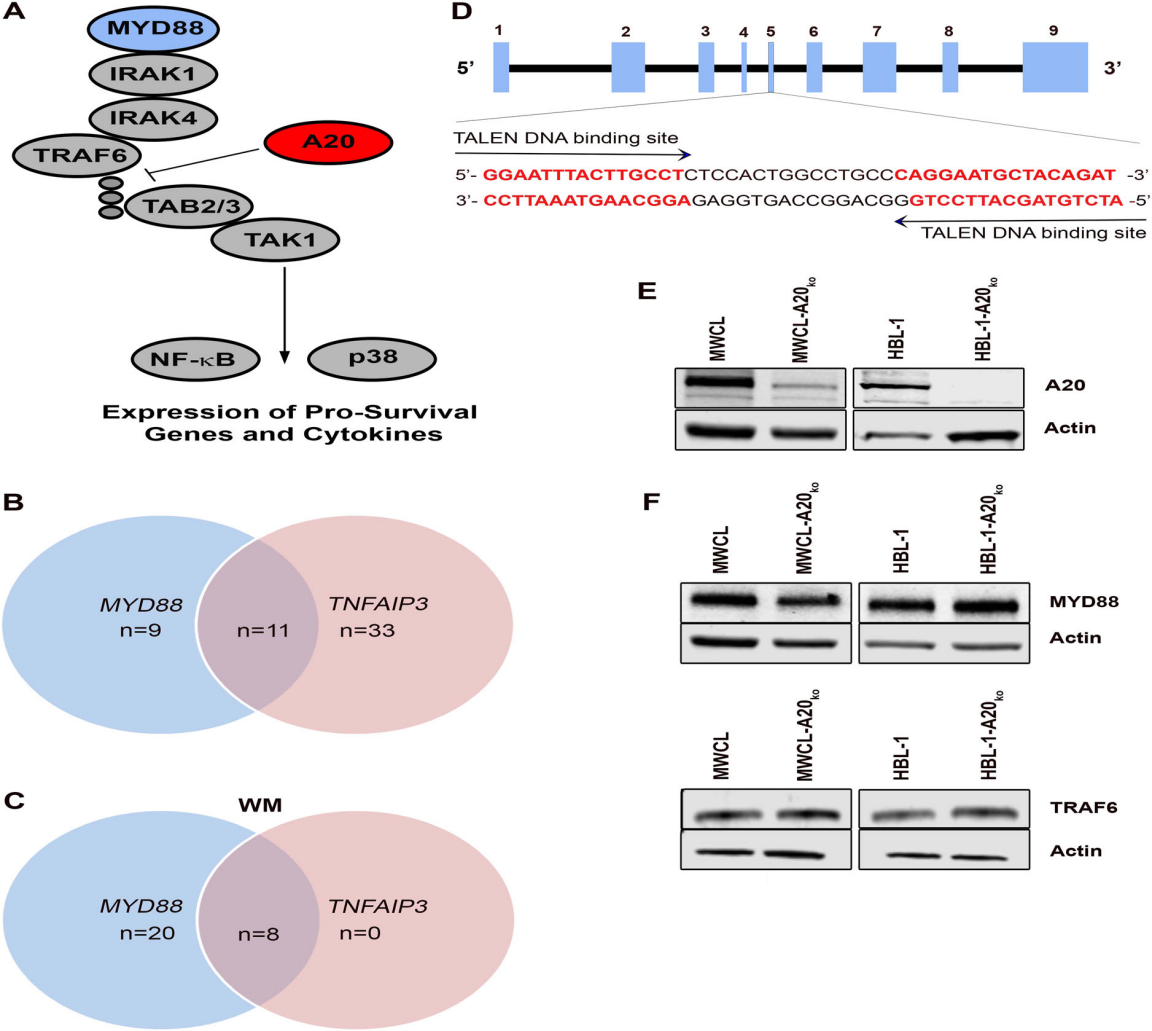
Frequency of *MYD88* mutations in combination with *TNFAIP3* genetic alterations in DLBCL and WM patients. **a** Schematic representation of the MYD88 signaling pathway. **b** Venn diagram of *MYD88* mutant and overlapping *TNFAIP3* loss in DLBCL patients. DLBCL *MYD88* mutation status was assessed by whole exome sequencing (WES) and *TNFAIP3* loss was determined by WES (*n* = 56) and OncoScan (*n* = 89) data. **c** Venn diagram of *MYD88* mutant and overlapping *TNFAIP3* loss in WM patients. *MYD88* status in WM patients was done by Sanger sequencing and *TNFAIP3* status was detected by gene copy number analysis. **d** Schematic representation of the genomic structure of the *TNFAIP3* gene. Exons are indicated by light blue boxes. TALENs were designed to target sequences in exon 5 and sequences of TALENs binding sites are highlighted in bold red. **e** Western blot analysis showed a reduction of *TNFAIP3* (A20) in the newly generated cell lines, compared to their wild type counterpart. β-Actin was used as a loading control (*n* = 3). **f** Western blot analysis of MYD88 and TRAF6 in MWCL and HBL-1 A20 knock out cell lines and their wild type counterpart. β-Actin was used as a loading control (*n* = 3)

Deubiquitinating enzymes such as A20 counteract E3-ligase activity, inhibit TRAF6 activity, and negatively regulate MYD88-driven TAK1, NF-κB, and p38 activation (Fig. 1a)^12,24^. To better understand the impact of *TNFAIP3* loss on *MYD88*_*L265P*_ in DLBCL and WM, we first wanted to determine the frequency of *MYD88* mutations in combination with *TNFAIP3* genetic alterations. Using genetic data from 145 cases of DLBCL, we found that 20 (13%) of the cases carried a *MYD88* mutation. 70% were ABC/non-GCB (nGCB)-DLBCL, 15% germinal center B cell DLBCL (GCB), and 15% unknown. Eleven of the 20 (55%) *MYD88* mutant DLBCL cases had a *TNFAIP3* loss (Fig. 1b). Taking a closer look at those cases, 73% had a *MYD88*_*L265P*_ mutation (ABC/nGCB-DLBCL, *n* = 5; GCB-DLBCL, *n* = 1; and unknown, *n* = 2) followed by 18% with *MYD88*_*S243N*_ (ABC/nGCB-DLBCL, *n* = 1 and GCB-DLBCL, *n* = 1), and 9% with *MYD88*_*M233T*_ (ABC/nGCB-DLBCL, *n* = 1). *TNFAIP3* loss was the most frequent (55%) copy number loss in our cohort of *MYD88* mutant cases (Supplemental Figure 2A). Other frequent losses in our *MYD88* mutant cases where *CDKN2A* (50%), *ARID1B* (45%), *ZNF292* (45%), and *PRDM1* (45%). Other genetic events that frequently occurred in combination with *MYD88* mutations included copy number gains of *BCL2* and *KLHL14* (45%) and mutations in *KMT2D* and *CD79B* (40%) (Supplemental Figure 2B and C). For our analysis of WM, we used genetic data from 29 cases and found that 97% (*n* = 28) carried a *MYD88* mutation. Of those, 28% (*n* = 8) had a *TNFAIP3* loss (Fig. 1c). The high incidence of *TNFAIP3* loss combined with *MYD88* mutations suggests a relationship between these genetic events. Statistical analyses of our data suggest that *TNFAIP3* loss and *MYD88* mutations are positively correlated in DLBCL (*p* = 0.017), but not WM (*p* = 1.0) WM, although our sample size was small. We were able to validate our findings in DLBCL using available data from the Chapuy et al.^25^ study which reported 6q or 6q23.3 loss and *MYD88* mutation in *n* = 304 patients (*p* < 0.0001). Together these data suggest that *TNFAIP3* loss is one of the most frequent genetic alterations in *MYD88* mutant DLBCL and WM.

To further understand the cellular consequence of *MYD88*_*L265P*_ in combination with *TNFAIP3* loss in human models of WM and DLBCL, we used TALENs genome editing technology to genetically modify the WM cell line MWCL and the ABC-DLBCL cell line HBL-1, both of which harbor a *MYD88*_*L265P*_ mutation, but have a wild type *TNFAIP3*. To introduce a *TNFAIP3* loss in those cell lines, we designed a unique pair of TALENs to target exon 5 of the *TNFAIP3* gene to induce a double strand break resulting in a base pair deletions in exon 5 (Fig. 1d). TALENs and a GFP co-expressing plasmid were transfected into the cell lines and GFP positive cells were single cell sorted, expanded, and screened for A20 loss by western blot (Fig. 1e). Western blot analysis showed 80% reduction of A20 in the WM cells and a nearly complete loss of A20 in the DLBCL cells, hereafter referred to as MWCL-A20_ko_ and HBL-1-A20_ko_, compared to their wild type counterpart. To ensure that A20 knockdown did not impact the expression of key MYD88 signaling molecules, we measured the expression of MYD88 and TRAF6 in each of the cell lines. We did not detect any changes in MYD88 or TRAF6 expression suggesting that the MYD88 signaling pathway is fully intact in MWCL-A20_ko_ and HBL-1-A20_ko_ cells (Fig. 1f) ensuring that signaling defects are specific for A20 loss.

### Loss of A20 enhances *MYD88*_*L265P*_ -driven signaling and contributes to ibrutinib resistance

**Fig. 2:**
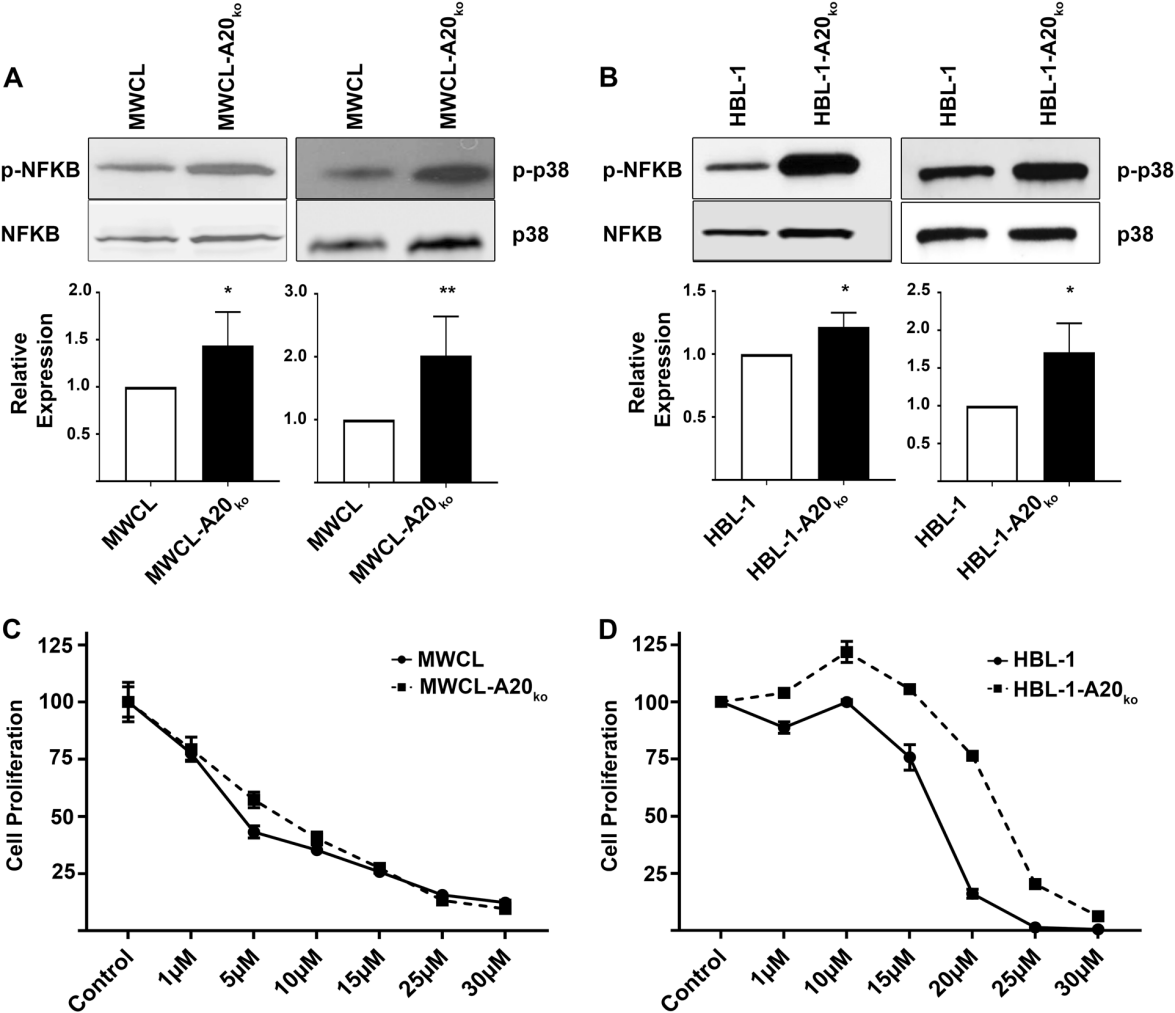
Increased baseline NF-κB and p38 phosphorylation in MWCL-A20_ko_ and HBL-1-A20_ko_ cells impacts ibrutinib response. **a** Western blot analysis of phosphorylated NF-κB in MWCL (*n* = 4) and HBL-1 (*n* = 3) A20 knock out cell lines and their wild type counterpart. Total NF-κB was used as a loading control. **b** Western blot analysis of phosphorylated p38 in MWCL (*n* = 5) and HBL-1 (*n* = 3) A20 knock out cell lines and their wild type counterpart. Total p38 was used as a loading control. Each bar represents the mean values of expression levels ± SD, *p ≤ 0.05, **p ≤ 0.01. **c**, **d** MWCL and HBL-1 cell lines were treated with indicated concentrations of ibrutinib or DMSO for 48 h and run in triplicate (*n* = 3). The representative independent experiment is shown and each point represents the mean normalized counts ± SD

To explore the possibility that loss of A20 activates *MYD88*_*L265P*_-driven signaling, we measured the impact of *TNFAIP3* deletion on activation of p38 and NF-κB in the MWCL-A20_ko_ and HBL-1-A20_ko_ cell lines. Western blot analysis revealed significant upregulation of phosphorylated p38 (2.02-fold in MWCL-A20_ko_, *p* = 0.006 and 1.71-fold in HBL-1-A20_ko_, *p* = 0.03) and NF-κB (1.44-fold in MWCL-A20_ko_, *p* = 0.05 and 1.22-fold in HBL-1-A20_ko_, *p* = 0.03) when compared to the wild type cell line controls (Fig. 2a, b). Graphical representation of multiple experiments is shown in the lower panel of each figure. These studies suggest that loss of A20 in human models of WM and DLBCL drives and enhanced *MYD88*_*L265P*_-driven signaling.

A recent study on DLBCL patients^17^ suggests that *TNFAIP3* inactivation negatively impacts therapeutic responses to ibrutinib, a BTK inhibitor known to inhibit B-cell receptor and NF-κB signaling^26,27^. Therefore, we next wanted to determine if ibrutinib responses were impacted by the loss of A20 in our cell models. The MWCL-A20_ko_ and HBL-1-A20_ko_ cell lines, along with their respective controls, were treated in a dose-dependent manner with ibrutinib and proliferation was measured after 48 h. We did not detect any significant differences in the response to ibrutinib in the WM cell lines (Fig. 2c, left panel). However, HBL-1-A20_ko_ was significantly (*p* < 0.05) more resistant to ibrutinib single agent therapy than HBL-1 wild type cell line (Fig. 2d, right panel). This data suggests that DLBCL patients with a *MYD88* mutation and an A20 loss are more resistant to ibrutinib single agent therapy.

### Loss of A20 induces upregulation of NF-κB target genes

**Fig. 3:**
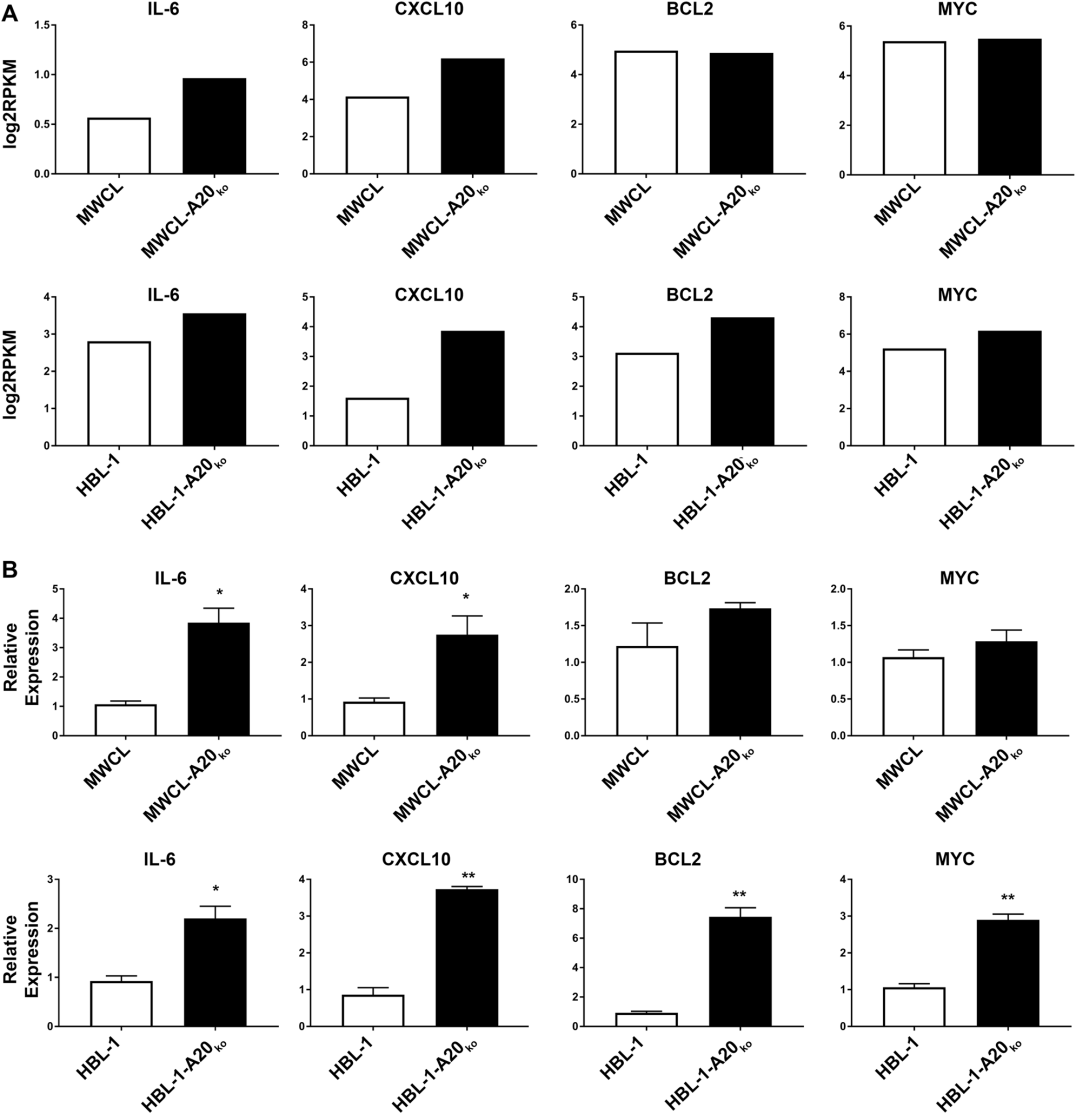
Upregulation of NF-κB Target gene RNA expression in MWCL-A20_ko_ and HBL-1-A20_ko_ cells. **a** Log(2) reads per kilobase per million mapped (RPKM) reads from RNASeq reveals up-regulation of the NF-κB target genes in MWCL-A20_ko_ and HBL-1-A20_ko_ cell lines (*n* = 1). **b** Bar graph representing fold increase of NF-κB target genes in MWCL-A20_ko_ (IL-6, *n* = 6; CXCL10, *n* = 5; BCL2, *n* = 3; MYC, *n* = 3) and HBL-1-A20_ko_ (IL-6, *n* = 5; CXCL10, *n* = 3; BCL2, *n* = 3; MYC, *n* = 3) cell lines determined by qPCR. The representative independent experiment is shown and all qPCR experiments were performed in duplicates. Each bar represents the mean values of expression levels ± SD. *p ≤ 0.05, **p ≤ 0.01

To further study the effects of A20 knock out, we performed RNASeq analysis of MWCL-A20_ko_, HBL-1-A20_ko_, and their matched control cell lines. The RNASeq data revealed that the NF-κB target genes, *IL-6*, *CXCL10*, *BCL2*, and *MYC* are upregulated in the A20 knock out cell lines (Fig. 3a). To validate the RNASeq findings, we performed quantitative PCR experiments on the cell lines and saw that *IL-6* and *CXCL10* were significantly upregulated in both MWCL-A20_ko_ and HBL-1-A20_ko_ compared to cells with intact *TNFAIP3* (Fig. 3b). *BCL2* and *MYC* were significantly upregulated in the HBL-1-A20_ko_ cells and showed a slight upregulation in the MWCL-A20_ko_. This data indicates that *TNFAIP3* loss drives upregulation of NF-κB target genes in DLBCL and WM.

### Upregulation of NF-κB target gene protein expression in A20 knock out cells

**Fig. 4:**
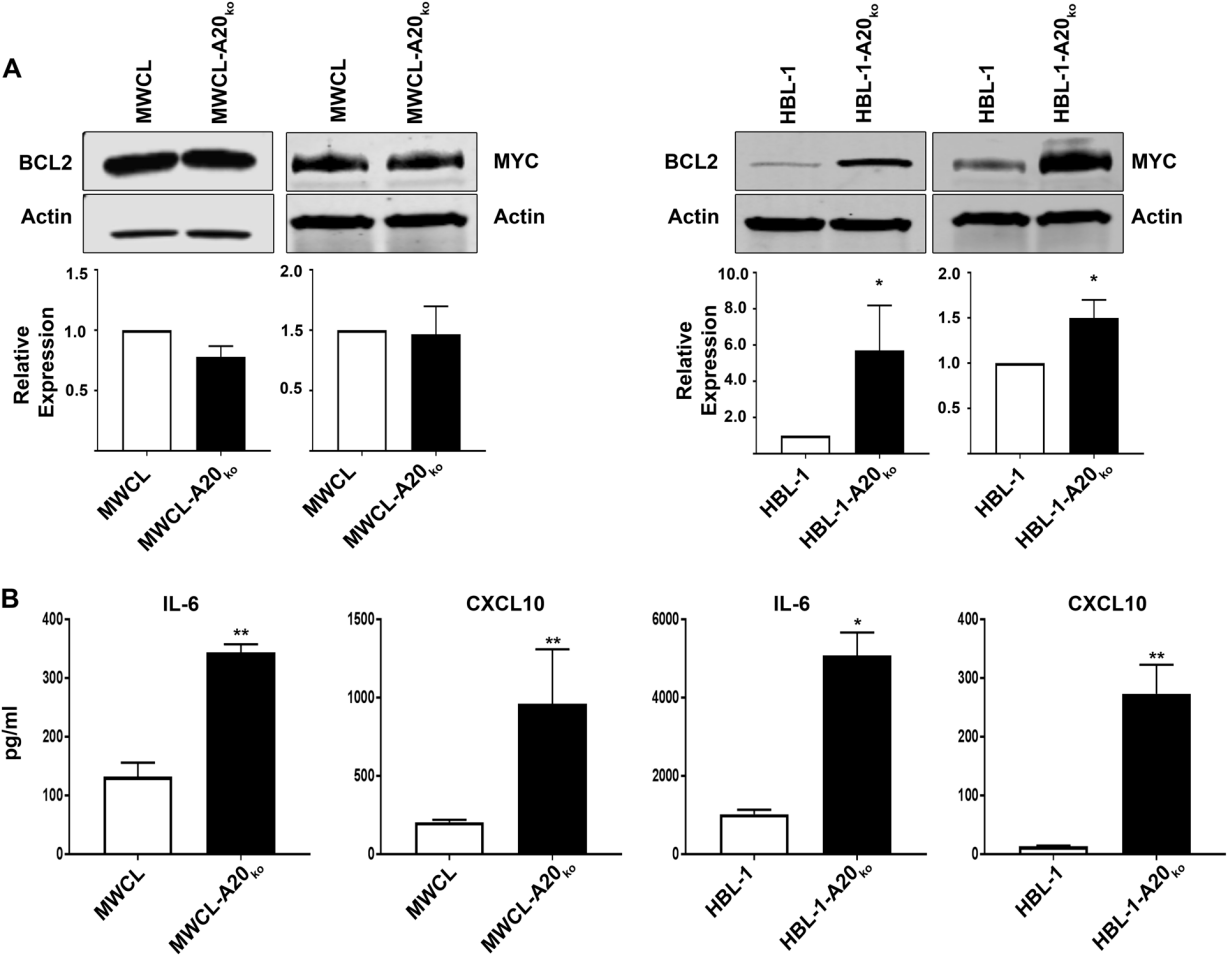
Upregulation of NF-κB target gene protein expression in MWCL-A20_ko_ and HBL-1-A20_ko_ cells. **a** Western blot analysis of NF-κB target genes BCL2 and MYC in MWCL (BCL2, *n* = 4; MYC, *n* = 3) and HBL-1 (BCL2, *n* = 3; MYC, *n* = 3) A20 knock out cell lines and their wild type counterpart β-actin was used as a loading control. **b** Upregulation of IL-6 and CXCL10 secretion in MWCL (*n* = 3) and HBL-1 (*n* = 3) A20 knock out cell lines and their wild type counterpart. The representative experiment is shown and cytokine assay was run in duplicates. Each bar represents the mean values of expression levels ± SD. *p ≤ 0.05, **p ≤ 0.01

To further validate RNA expression data, we performed western blot analysis of BCL2 and MYC (Fig. 4a) and confirmed that both are significantly upregulated in the HBL-1-A20_ko_ cell line. Graphical representation of multiple experiments is shown in the lower panel of each figure. There were no changes in the protein levels of BCL2 and MYC in the MWCL-A20_ko_ suggesting that loss of A20 in DLBCL and WM may have different biologic effect in these two forms of NHL. Another possibility that may explain the differences in BCL2 and MYC protein upregulation in our knock out cell lines is the residual A20 protein expression found in the MWCL-A20_ko_ compared to the HBL-1 cells. Therefore, we generated a second WM cell line with A20 loss (BCWM-A20_ko_) that has a 94% reduction in A20 expression (Supplemental Figure 3A). Again, we did not detect any changes in BCL2 or MYC (*p* = 0.1104 and *p* = 0.7624, *n* = 3) protein expression in the BCWM-A20_ko_ compared to the control cells (Supplement Figure 3B and C). This data further supports the idea that A20 loss has differential effects in WM and DLBCL, which may be expected due to disease heterogeneity.

To assess the expression of IL-6 and CXCL10, we carried out Luminex single plex assays (Fig. 4b). Cells were cultured for 48 h in standard media and supernatants were analyzed. Both IL-6 and CXCL10 were significantly upregulated in the media of MWCL-A20_ko_ and HBL-1-A20_ko_ cell lines compared to their wild type counterpart (Fig. 4b). These data validate our previous findings and indicate that loss of A20 drives expression of BCL2, MYC, IL-6, and CXCL10.

### *MYD88*_*L265P*_ and *TNFAIP3* loss drives the JAK/STAT pathway

**Fig. 5:**
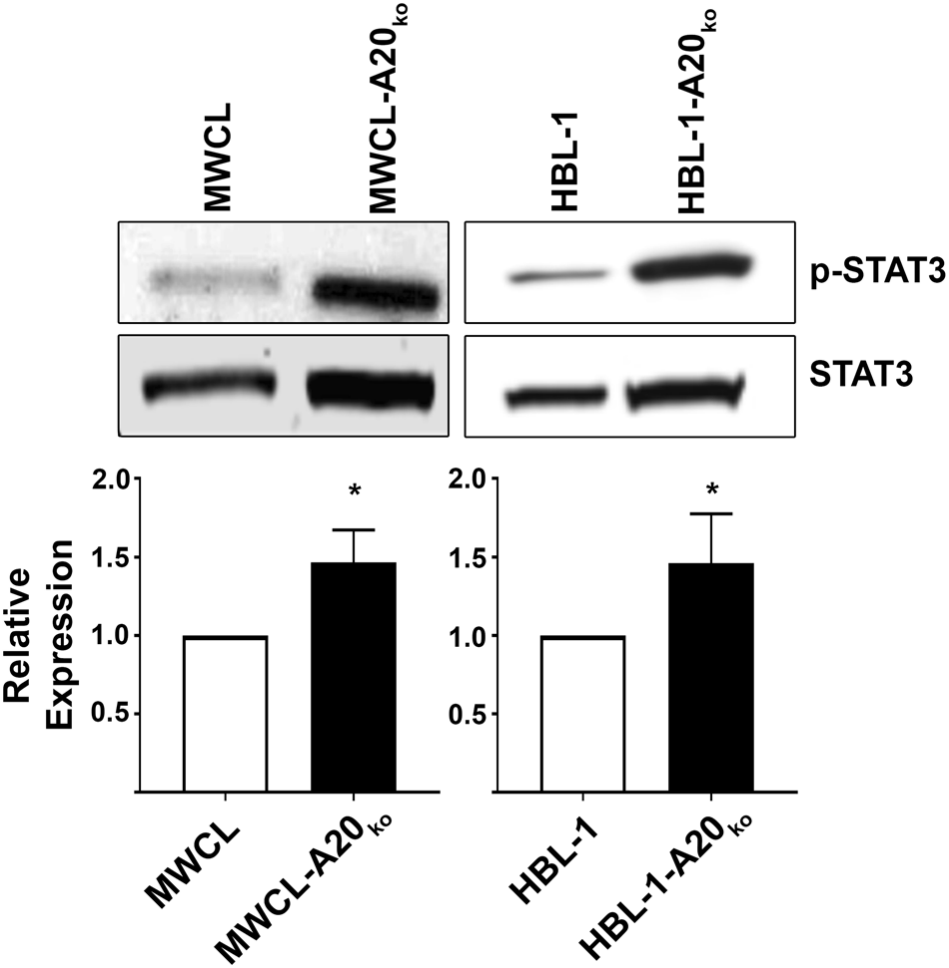
Increased baseline STAT3 phosphorylation in MWCL-A20_ko_ and HBL-1-A20_ko_ cells. Western blot analysis of phosphorylated STAT3 in MWCL (*n* = 3) and HBL-1 (*n* = 5) A20 knock out cell lines and their wild type counterpart. Total STAT3 was used as a loading control. Each bar represents the mean values of expression levels ± SD. *p ≤ 0.05, **p ≤ 0.01

MYD88_L265P_ has been shown to drive autocrine expression of cytokines resulting in the activation of the JAK/STAT pathway^2,28^. Therefore, we next sought to determine if our newly generated A20 knock out cell lines had increased STAT3 activation, a downstream target of IL-6. Western blot analysis of phosphorylated STAT3 levels showed that MWCL-A20_ko_ and HBL-1-A20_ko_ cell lines had significantly higher levels of p-STAT3 (1.47-fold in MWCL-A20_ko_, *p* = 0.0176 and 1.46-fold in HBL-1-A20_ko_, *p* = 0.0114) (Fig. 5). Graphical representation of multiple experiments is shown in the lower panel. These data suggest that upregulation of IL-6 induced by A20 loss drives activation of the JAK/STAT in WM and DLBCL cells.

## Discussion

In this study, we sought to better define the genetic profile and biologic impact of *MYD88* mutations in combination with *TNFAIP3* loss in WM and DLBCL. *MYD88*_*L265P*_ drives cell proliferation, but it is rapidly shut down by a *TNFAIP3* negative feedback loop, suggesting an important relationship between these proteins^8^. Our data show that a large percentage of DLBCL and WM cases that have a *MYD88* mutation also harbor a *TNFAIP3* loss, 55% DLBCL and 28%, respectively. These data are supported by previously published work showing that 11–55% of the *MYD88* mutant DLBCL have a *TNFAIP3* genetic alteration^2,29,30^ and 35–50% of WM patients harbor a *TNFAIP3* loss^31,32^. The importance of gaining insight of the impact of *MYD88* mutations in combination with other genomic events in lymphoma is further supported by recent findings showing a correlation between *MYD88* mutation status and poor outcome in DLBCL^33^. Furthermore, in the study performed by Reddy et al. *MYD88* mutations alone are a prognostic factor for the ABC-DLBCL subgroup^29^. However, additional studies counter these data and suggest a need for additional genetic and clinical analysis to further define the impact by *MYD88* mutations in combination with other genetic alterations^30,34–36^. Two recent large-scale studies shed new insight on how genomic alterations contribute to overall survival in DLBCL patients^25,37^. Both analyses define novel and genomically unique subgroups of patients with inferior overall survival, MCD^37^ and C5^25^. Both MCD and C5 share genetic enrichment for *MYD88* and *CD79B* mutations. In the MCD classification, 2.9% of the patients also have a *TNFAIP3* loss, where 46% of *MYD88* mutant patients in the C5 group either have a 6q or 6q23.3 loss. Between these studies, it is not clear why there is such a large discrepancy in the rate of *TNFAIP3* loss in the *MYD88* mutant cases, although our study is in line with the 46% described by Chapuy et al. as well as other publications^25,30^. This variability is most likely due to the use of different copy number analysis platforms as well as tissue samples type (i.e., fresh frozen vs FFPE). Taken together, this data supports the possibility that loss of *TNFAIP3* contributes to the *MYD88* effect on outcome and future studies should further evaluate the clinical significance.

Using TALEN genome-editing technology, we were able to generate cell lines that allow for functional characterization of causal genetic variants. One benefit to genome editing of cell lines is that it allows for a direct comparison of a specific alteration in the same genetic background. This system also permitted us to design a genetic model in both DLBCL and WM cell lines, allowing for validation of our results across multiple cell lines. Using the MWCL-A20_ko_ and HBL-1-A20_ko_, we were able to show that loss of *TNFAIP3* enhances *MYD88*_*L265P*_-driven NF-κB and p38 signaling resulting in increased expression of NF-κB target genes *IL-6* and *CXCL10*, known NF-κB target genes^2,32,38,39^, have both been shown to be significantly upregulated in WM and DLBCL, and higher serum levels correlate with an inferior survival^38,40–43^. Interestingly, in a new T-cell specific A20 knock out model, serum levels of CXCL10 were significantly increased supporting the idea that CXCL10 expression is regulated by A20^44^. The role of CXCL10 in B cell lymphomas has not been studied well, but it is known that its receptor CXCR3 is expressed on a small subset of B cells^45,46^. Additionally, we show that *TNFAIP*3 loss contributes to ibrutinib resistance in an ABC-DLBCL cell line. This data aligns with other studies showing that genetic aberrations contribute to ibrutinib resistance in hematological malignancies^47–50^. A recent study by Kuo et al. showed that ibrutinib resistance is marked by BCL2 upregulation and that combining ibrutinib with ABT-199, a BCL2 inhibitor, could overcome resistance in DLBCL^51^. Together, these studies suggest that genetic analysis of tumors may inform therapeutic choices and highlights the potential importance of individualized therapy based on genetic profiles.

IL-6 regulation by A20 has been shown in mouse models where *TNFAIP3* was depleted in B cells. Mice lacking A20 had higher mRNA levels and secreted more IL-6 than mice having an intact *TNFAIP3* locus after B cell activation^52,53^. Gene expression analysis of WM patients has shown that *IL-6* is one of the most increased expressed genes and A20 is lost in a high percentage of WM patients indicating that A20 controls cytokine production in WM^31,40^. A previous study identified a subgroup of ABC-DLBCL with a high autocrine IL-6 production driving phosphorylation of STAT3. *MYD88* mutations were highly enriched in this subgroup showing that IL-6 production is driven by dysregulated MYD88 signaling^2,28^. However, our data indicate that this dysregulation can be further driven by *TNFAIP3* loss in patients with *MYD88* mutations.

The importance of IL-6 and the autocrine mechanism by which IL-6 induces STAT3 activation in DLBCL has been shown in previous studies^28,54^. STAT3 overexpression is a prognostic marker for overall survival in DLBCL and high STAT3 and phosphorylated STAT3 in the nucleus correlates with inferior survival on those patients^55,56^. Furthermore, it has been shown that STAT3 upregulation contributes to IgM secretion in WM, which can cause severe complication in WM patients^57,58^. A recent study demonstrated how STAT3 acts as an activator of several oncogenic pathways as well as a suppressor for apoptosis^59^. Additionally, it has been shown that MYC and BCL2 overexpression significantly correlates with high phosphorylation of STAT3 in DLBCL^60^. Our data support these findings and extend to our mechanistic understanding of how MYD88 and A20 signaling can contribute to IL-6 secretion, STAT3 activation, and expression of pro-survival genes.

*BCL2* and *MYC* are both well-studied oncogenes and are targets of recurrent chromosomal breakpoints in lymphomas^61,62^. If both *MYC* and *BCL2* undergo rearrangement at the same time, they are referred to as double hit lymphoma, and often fall into the GCB-DLBCL subgroup^63–66^. However, there is also another subclass which is referred to as double expressers, where MYC and BCL2 are overexpressed independent of genetic rearrangement, and often fall into the ABC-DLBCL subgroup^67^. A recent study showed that BCL2 and MYC expression is significantly associated with *MYD88* mutations in the ABC-DLBCL subgroup, however this study only looked at the *MYD88* mutation status and no other genetic alterations in combination to *BCL2* and *MYC* status^30^. Our data indicate that *TNFAIP3* loss together with *MYD88*_*L265P*_ drives upregulation of anti-apoptotic and cell survival signaling in DLBCL. It would be interesting to see if double expressing ABC-DLBCL also harbor a *MYD88* mutation or *TNFAIP3* loss or both together since our data indicate that both genetic aberrations contribute to *BCL2* and *MYC* expression in ABC-DLBCL. On the other hand, recent studies have shown that expression levels of the *BCL2* family in WM patients is almost the same as in healthy controls and that WM cell lines treated with ABT-737, a BCL2 inhibitor, lack sensitivity^68,69^. Together, this data supports the idea that the biological pathways activated by *MYD88* and *TNFAIP3* loss in WM and DLBCL are unique. In summary, we have established a new WM and DLBCL cell line model that mimics the effect of the *MYD88*_*L265P*_ mutation in combination with a loss of the *TNFAIP3* gene and A20 expression. We show that loss of *TNFAIP3* results in a higher baseline phosphorylation of NF-κB, p38, and STAT3. Additionally, loss of *TNFAIP3* impacts expression of IL-6 and CXCL10. Overall, results from this study contribute to our understanding of *MYD88*-driven lymphomas, suggests a possible clinical implication for those individuals that harbor both a *MYD88* mutation and a loss of *TNFAIP3*, and also provides us with a useful model to study novel therapeutic strategies in patients who harbor these genetic variants.

## Electronic supplementary material

Supplemental Figures

Supplemental Methods

## References

[1] Forbes SA (2017). COSMIC: somatic cancer genetics at high-resolution. Nucleic Acids Res..

[2] Ngo VN (2011). Oncogenically active MYD88 mutations in human lymphoma. Nature.

[3] Treon SP (2012). MYD88 L265P somatic mutation in Waldenstrom’s macroglobulinemia. N. Engl. J. Med..

[4] Braggio E (2015). Genome-wide analysis uncovers novel recurrent alterations in primary central nervous system lymphomas. Clin. Cancer Res..

[5] Salcedo R, Cataisson C, Hasan U, Yuspa SH, Trinchieri G (2013). MyD88 and its divergent toll in carcinogenesis. Trends Immunol..

[6] Landgren O, Tageja N (2014). MYD88 and beyond: novel opportunities for diagnosis, prognosis and treatment in Waldenstrom’s macroglobulinemia. Leukemia.

[7] Ansell SM (2014). Activation of TAK1 by MYD88 L265P drives malignant B-cell growth in non-Hodgkin lymphoma. Blood Cancer J..

[8] Wang JQ, Jeelall YS, Beutler B, Horikawa K, Goodnow CC (2014). Consequences of the recurrent MYD88(L265P) somatic mutation for B cell tolerance. J. Exp. Med..

[9] Knittel G (2016). B-cell-specific conditional expression of Myd88p.L252P leads to the development of diffuse large B-cell lymphoma in mice. Blood.

[10] Compagno M (2009). Mutations of multiple genes cause deregulation of NF-kappaB in diffuse large B-cell lymphoma. Nature.

[11] Braggio E (2009). Identification of copy-number abnormalities and inactivating mutations in two negative regulators of NF-kB signaling pathways in Waldenström’s macroglobulinemia. Cancer Res..

[12] Ma A, Malynn BA (2012). A20: linking a complex regulator of ubiquitylation to immunity and human disease. Nat. Rev. Immunol..

[13] Kato M (2009). Frequent inactivation of A20 in B-cell lymphomas. Nature.

[14] Schmitz R (2009). TNFAIP3 (A20) is a tumor suppressor gene in Hodgkin lymphoma and primary mediastinal B cell lymphoma. J. Exp. Med..

[15] Kelly PN (2015). Selective interleukin-1 receptor–associated kinase 4 inhibitors for the treatment of autoimmune disorders and lymphoid malignancy. J. Exp. Med..

[16] Booher RN, Samson ME, Xu GX, Cheng H, Tuck DP (2017). Abstract 1168: Efficacy of the IRAK4 inhibitor CA-4948 in patient-derived xenograft models of diffuse large B cell lymphoma. Cancer Res..

[17] Wilson WH (2015). Targeting B cell receptor signaling with ibrutinib in diffuse large B cell lymphoma. Nat. Med..

[18] Lohr JG (2012). Discovery and prioritization of somatic mutations in diffuse large B-cell lymphoma (DLBCL) by whole-exome sequencing. Proc. Natl Acad. Sci. USA.

[19] Hans CP (2004). Confirmation of the molecular classification of diffuse large B-cell lymphoma by immunohistochemistry using a tissue microarray. Blood.

[20] Wright G (2003). A gene expression-based method to diagnose clinically distinct subgroups of diffuse large B cell lymphoma. Proc. Natl Acad. Sci. USA.

[21] Scott DW (2014). Determining cell-of-origin subtypes of diffuse large B-cell lymphoma using gene expression in formalin-fixed paraffin-embedded tissue. Blood.

[22] Hodge LS (2011). Establishment and characterization of a novel Waldenstrom macroglobulinemia cell line, MWCL-1. Blood.

[23] Ma AC (2016). FusX: a rapid one-step transcription activator-like effector assembly system for genome science. Hum. Gene Ther..

[24] Vereecke L, Beyaert R, van Loo G (2009). The ubiquitin-editing enzyme A20 (TNFAIP3) is a central regulator of immunopathology. Trends Immunol..

[25] Chapuy B (2018). Molecular subtypes of diffuse large B cell lymphoma are associated with distinct pathogenic mechanisms and outcomes. Nat. Med..

[26] Herman SE (2014). Ibrutinib inhibits BCR and NF-kappaB signaling and reduces tumor proliferation in tissue-resident cells of patients with CLL. Blood.

[27] Rushworth SA (2013). BTK inhibitor ibrutinib is cytotoxic to myeloma and potently enhances bortezomib and lenalidomide activities through NF-κB. Cell Signal..

[28] Lam LT (2008). Cooperative signaling through the signal transducer and activator of transcription 3 and nuclear factor-κB pathways in subtypes of diffuse large B-cell lymphoma. Blood.

[29] Reddy A (2017). Genetic and functional drivers of diffuse large B cell lymphoma. Cell.

[30] Dubois S (2017). Biological and clinical relevance of associated genomic alterations in MYD88 L265P and non-L265P-mutated diffuse large B-cell lymphoma: analysis of 361 cases. Clin. Cancer Res..

[31] Hunter ZR (2014). The genomic landscape of Waldenström macroglobulinemia is characterized by highly recurring MYD88 and WHIM-like CXCR4 mutations, and small somatic deletions associated with B-cell lymphomagenesis. Blood.

[32] Poulain S (2013). MYD88 L265P mutation in Waldenstrom macroglobulinemia. Blood.

[33] Fernandez-Rodriguez C (2014). MYD88 (L265P) mutation is an independent prognostic factor for outcome in patients with diffuse large B-cell lymphoma. Leukemia.

[34] Treon SP (2014). Somatic mutations in MYD88 and CXCR4 are determinants of clinical presentation and overall survival in Waldenstrom macroglobulinemia. Blood.

[35] Abeykoon JP (2018). MYD88 mutation status does not impact overall survival in Waldenstrom macroglobulinemia. Am. J. Hematol..

[36] Yu S (2018). High frequency and prognostic value of MYD88 L265P mutation in diffuse large B-cell lymphoma with R-CHOP treatment. Oncol. Lett..

[37] Schmitz R (2018). Genetics and pathogenesis of diffuse large B-cell lymphoma. N. Engl. J. Med..

[38] Yang G (2016). HCK is a survival determinant transactivated by mutated MYD88, and a direct target of ibrutinib. Blood.

[39] Harris DP, Bandyopadhyay S, Maxwell TJ, Willard B, DiCorleto PE (2014). Tumor necrosis factor (TNF)-alpha induction of CXCL10 in endothelial cells requires protein arginine methyltransferase 5 (PRMT5)-mediated nuclear factor (NF)-kappaB p65 methylation. J. Biol. Chem..

[40] Chng WJ (2006). Gene-expression profiling of Waldenstrom macroglobulinemia reveals a phenotype more similar to chronic lymphocytic leukemia than multiple myeloma. Blood.

[41] Elsawa SF (2011). Comprehensive analysis of tumor microenvironment cytokines in Waldenstrom macroglobulinemia identifies CCL5 as a novel modulator of IL-6 activity. Blood.

[42] Ansell SM (2012). Elevated pretreatment serum levels of interferon-inducible protein-10 (CXCL10) predict disease relapse and prognosis in diffuse large B-cell lymphoma patients. Am. J. Hematol..

[43] Nacinovic-Duletic A, Stifter S, Dvornik S, Skunca Z, Jonjic N (2008). Correlation of serum IL-6, IL-8 and IL-10 levels with clinicopathological features and prognosis in patients with diffuse large B-cell lymphoma. Int. J. Lab. Hematol..

[44] Giordano M (2014). The tumor necrosis factor alpha-induced protein 3 (TNFAIP3, A20) imposes a brake on antitumor activity of CD8 T cells. Proc. Natl Acad. Sci. USA.

[45] Jones D, Benjamin RJ, Shahsafaei A, Dorfman DM (2000). The chemokine receptor CXCR3 is expressed in a subset of B-cell lymphomas and is a marker of B-cell chronic lymphocytic leukemia. Blood.

[46] Nanki T (2009). Chemokine receptor expression and functional effects of chemokines on B cells: implication in the pathogenesis of rheumatoid arthritis. Arthritis Res. Ther..

[47] Woyach JA (2014). Resistance mechanisms for the Bruton’s tyrosine kinase inhibitor ibrutinib. N. Engl. J. Med..

[48] Liu TM (2015). Hypermorphic mutation of phospholipase C, γ2 acquired in ibrutinib-resistant CLL confers BTK independency upon B-cell receptor activation. Blood.

[49] Xu L (2017). Acquired mutations associated with ibrutinib resistance in Waldenstrom macroglobulinemia. Blood.

[50] Barretina J (2012). The Cancer Cell Line Encyclopedia enables predictive modelling of anticancer drug sensitivity. Nature.

[51] Kuo HP (2017). Combination of ibrutinib and ABT-199 in diffuse large B-cell lymphoma and follicular lymphoma. Mol. Cancer Ther..

[52] Tavares RM (2010). The ubiquitin modifying enzyme A20 restricts B cell survival and prevents autoimmunity. Immunity.

[53] Chu Y (2011). B cells lacking the tumor suppressor TNFAIP3/A20 display impaired differentiation and hyperactivation and cause inflammation and autoimmunity in aged mice. Blood.

[54] Ding BB (2008). Constitutively activated STAT3 promotes cell proliferation and survival in the activated B-cell subtype of diffuse large B-cell lymphomas. Blood.

[55] Huang X (2013). Activation of the STAT3 signaling pathway is associated with poor survival in diffuse large B-cell lymphoma treated with R-CHOP. J. Clin. Oncol..

[56] Wu ZL, Song YQ, Shi YF, Zhu J (2011). High nuclear expression of STAT3 is associated with unfavorable prognosis in diffuse large B-cell lymphoma. J. Hematol. Oncol..

[57] Hodge LS (2012). IL-21 in the bone marrow microenvironment contributes to IgM secretion and proliferation of malignant cells in Waldenstrom macroglobulinemia. Blood.

[58] Gertz MA, Fonseca R, Rajkumar SV (2000). Waldenström’s macroglobulinemia. Oncologist.

[59] Lu L (2018). Gene regulation and suppression of type I interferon signaling by STAT3 in diffuse large B cell lymphoma. Proc. Natl Acad. Sci. USA.

[60] Ok CY (2014). Clinical implications of phosphorylated STAT3 expression in de novo diffuse large B-cell lymphoma. Clin. Cancer Res..

[61] Yip KW, Reed JC (2008). Bcl-2 family proteins and cancer. Oncogene.

[62] Dang CV (2012). MYC on the path to cancer. Cell.

[63] Weiss LM, Warnke RA, Sklar J, Cleary ML (1987). Molecular analysis of the t(14;18) chromosomal translocation in malignant lymphomas. N. Engl. J. Med..

[64] Kramer MH (1998). Clinical relevance of BCL2, BCL6, and MYC rearrangements in diffuse large B-cell lymphoma. Blood.

[65] Johnson NA (2009). Lymphomas with concurrent BCL2 and MYC translocations: the critical factors associated with survival. Blood.

[66] Kluin, P. M. B-cell lymphoma, unclassifiable, with features intermediate between diffuse large B-cell lymphoma and Burkitt lymphoma. *WHO Classification of Tumors of Haematopoietic and Lymphoid Tissues*. International Agency for Research on Cancer, 69008 Lyon, France, 265–266 (2008).

[67] Hu S (2013). MYC/BCL2 protein coexpression contributes to the inferior survival of activated B-cell subtype of diffuse large B-cell lymphoma and demonstrates high-risk gene expression signatures: a report from The International DLBCL Rituximab-CHOP Consortium Program. Blood.

[68] Gaudette BT (2016). Low expression of pro-apoptotic Bcl-2 family proteins sets the apoptotic threshold in Waldenström Macroglobulinemia. Oncogene.

[69] Chitta KS (2013). Heterogeneous Bcl-2 family expression In Waldenström Macroglobulinemia determines response to inducers of intrinsic apoptosis. Blood.

