# Supplementary material for: Loss of TNFAIP3 enhances MYD88L265P-driven signaling in non-Hodgkin lymphoma
Source: Blood Cancer J. 2018 Oct 9;8(10):97. doi: 10.1038/s41408-018-0130-3 (PMC6177394; doi:10.1038/s41408-018-0130-3)
Supplement: Supplementary file 1 — Supplemental Figures [file 41408_2018_130_MOESM1_ESM.doc]

**Supplemental Figures**

**Figure S1**


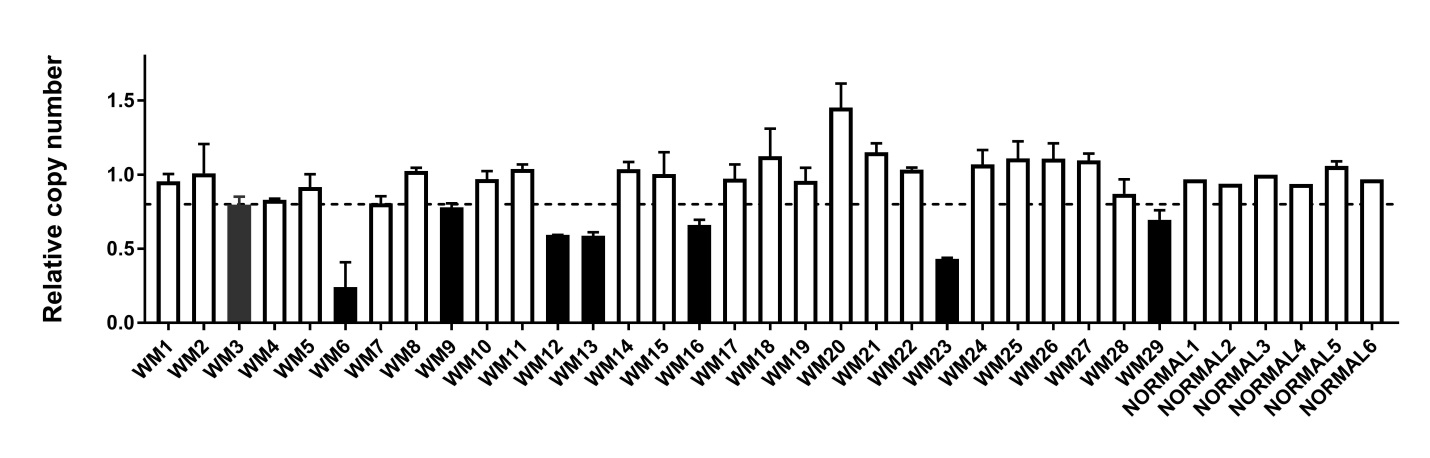


**Figure S1.** **Gene copy number analysis of A20 in WM patients.** 29 WM samples have been analyzed for *TNFAIP3* loss by using the TaqMan bases copy number assay. Cut off for *TNFAIP3* loss is corresponding to four times the SD of the mean value of all normal controls – denoted as the black line- and was set at 0.8. White bar graphs represent WM patients with intact *TNFAIP3* gene were black bar graphs represent patients with a *TNFAIP3* loss. Experiments were performed three times for each patient sample and the bars represent the mean values of expression levels ± SD

**Figure S2**


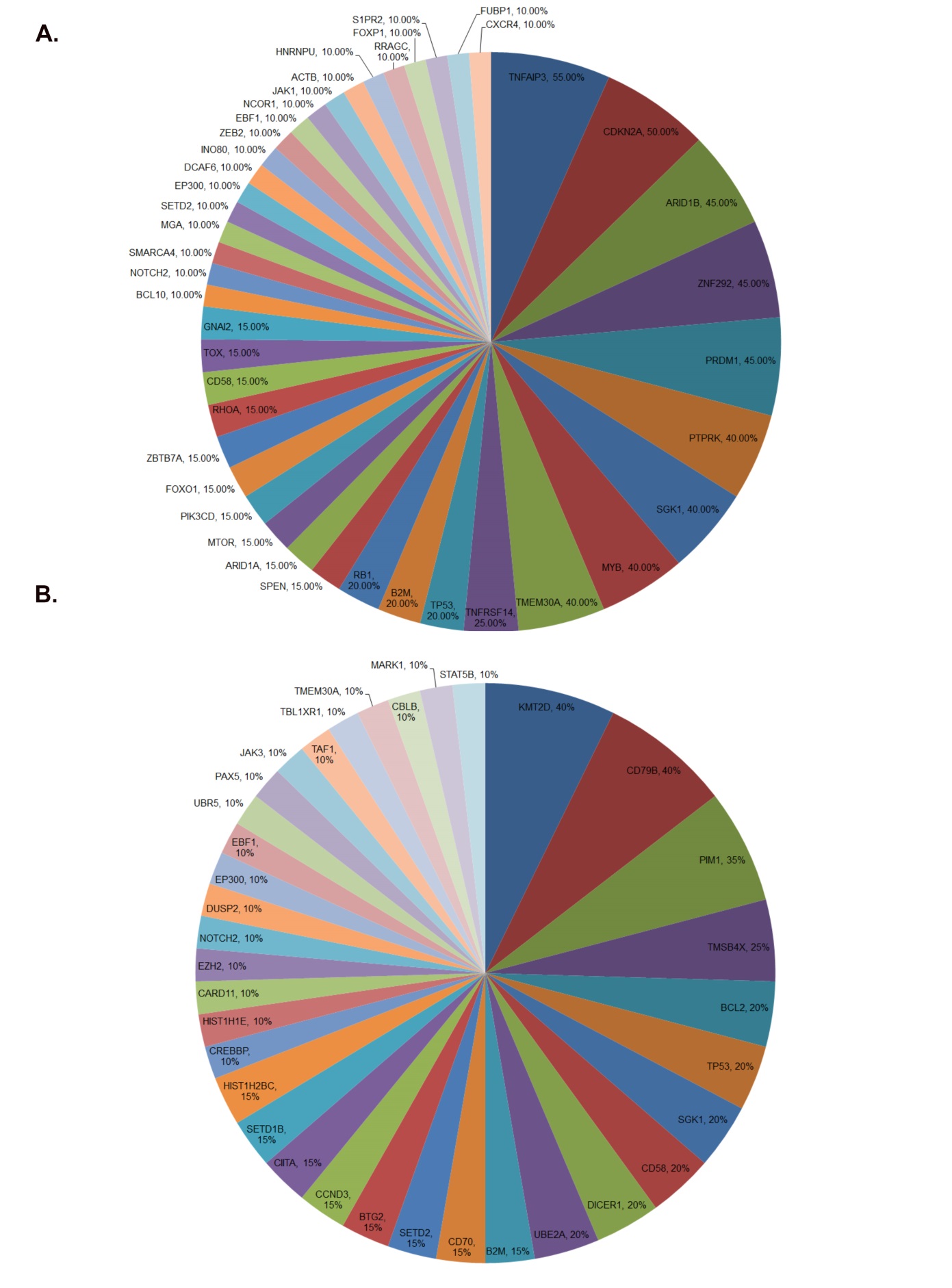


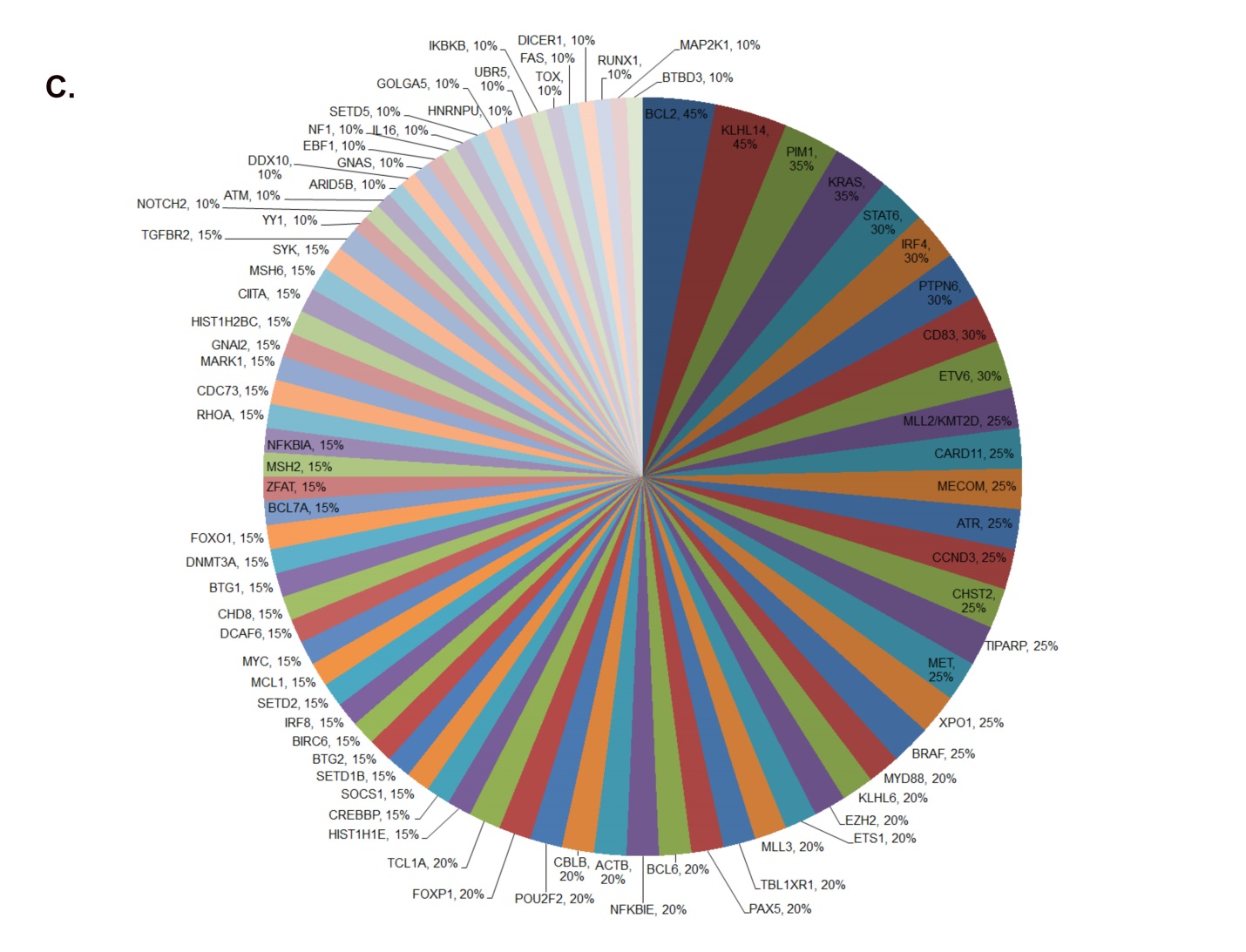


**Figure S2.** **Overview of genetic aberrations in *MYD88* mutated DLBCL**. A. Frequency of copy number loss which are found in *MYD88* mutated DLBCL. B. Most common mutations co-occurring with *MYD88* mutations in DLBCL in our cohort (n=20). C. Frequency of copy number gains found in *MYD88* mutated DLBCL. Cut off for this analysis was that the genetic event has to be found in at least 10% of the *MYD88* mutated cases. Genes considered for this analysis (n=150) were reported by *Reddy et al.* (1).

**Figure S3**


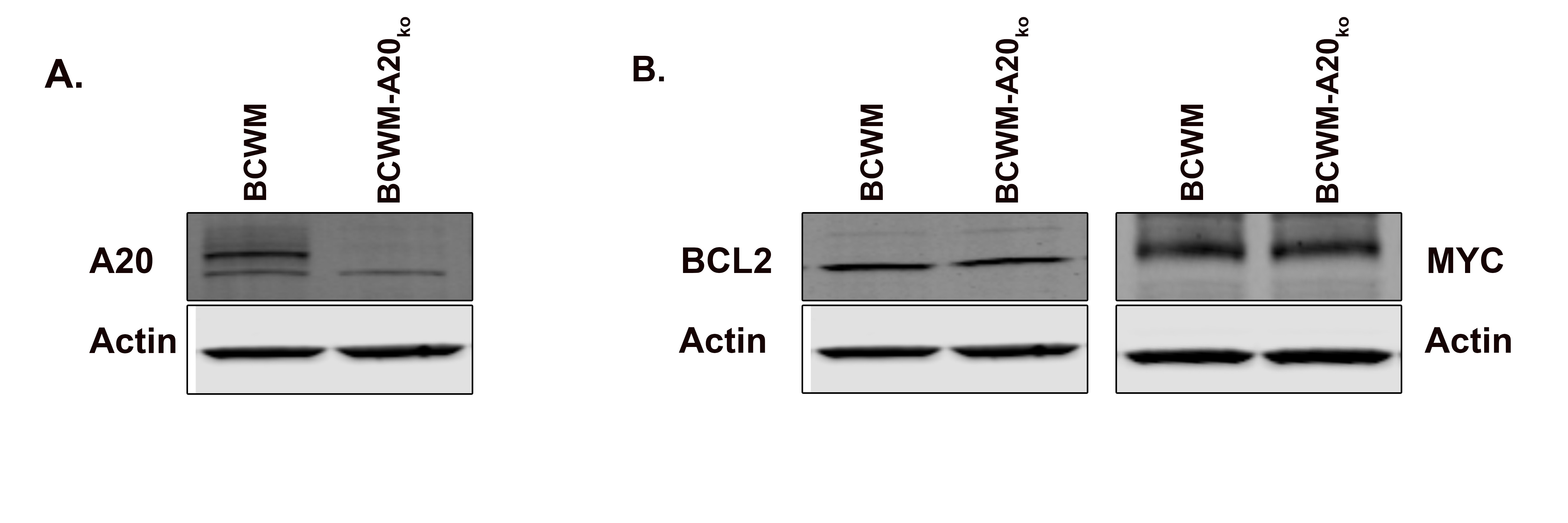


**Figure S3. Generation of BCWM Cells With a *TNFAIP3* Loss.** A. Western blot analysis of A20 in BCWM and BCWM-A20_ko_ cell lines. β-Actin was used as a loading control B. Western blot analysis of BCL2 and MYC in BCWM and BCWM-A20_ko_ cell lines. β-Actin was used as a loading control. Images shown are representative of n=3 experiments.

1. Reddy A, Zhang J, Davis NS, Moffitt AB, Love CL, Waldrop A, et al. Genetic and Functional Drivers of Diffuse Large B Cell Lymphoma. Cell. 2017;171(2):481-94.e15.
