# Supplementary material for: Loss of TNFAIP3 enhances MYD88L265P-driven signaling in non-Hodgkin lymphoma
Source: Blood Cancer J. 2018 Oct 9;8(10):97. doi: 10.1038/s41408-018-0130-3 (PMC6177394; doi:10.1038/s41408-018-0130-3)
Supplement: Supplementary file 2 — Supplemental Methods [file 41408_2018_130_MOESM2_ESM.docx]

**Supplemental Methods**

**DNA extraction from FFPE slides**

DNA was extracted from 89 de novo DLBCL tumors in FFPE using the QIAamp DNA FFPE Tissue Kit (Qiagen GmbH, Hilden, Germany) in the Mayo Biospecimens Accessioning and Processing Core. Prior to isolation, tumor blocks were reviewed by a Mayo Clinic hematopathologist, tumor areas were circled, and four 1 mm cores were used for DNA isolation. The minimum tumor purity for study was 30%. DNA quantity was measured using the Qubit Fluorometer (Thermo Scientific, Waltham, MA, USA) instrument.

**Whole exome analysis**

WES of tumor normal pairs (n=56) was performed as previously described (1, 2). WES of DNA extracted from FFPE (n=89) was performed at the Mayo Clinic Genome Analysis Core. Sequencing was carried out on an Illumina HiSeq 2000 at a depth of ~100 million 100 bp paired-end reads per sample. Data from all cases (n=145) were mapped to human genome reference build 37 using BWA-MEM (3). Quality control was performed by FASTQC (v0.11.3). After realignment and recalibration by GATK (v3.4-46), SNV and INDELs from individual germline and tumor samples were called by GATK haplotype caller (v3.4-46). Somatic mutations were defined as those not present in the 56 paired normal samples, and with population frequencies < 1% in HapMap, 1000 Genome Project, and in ESP (the Exome Sequencing Project, https://esp.gs.washington.edu/drupal/). Variants were annotated using the Mayo Clinic in-house annotation tool BioR (4).

**Copy number analysis**

56 of the case were analyzed for copy number alterations using raw WES files. The remaining cases (n=89) were processed at the Mayo Clinic Cytogenetics Lab using the molecular inversion probe OncoScan™ FFPE Assay Kit (Affymetrix, Santa Clara, CA, USA). Raw WES and OSCHP files were analyzed using Nexus Copy Number 9.0 software (Biodiscovery, El Segundo, USA). Data interpretation and copy number calling was done using the human reference genome GRCh37/hg19. Files were analyzed using the Nexus FASST2-Segmentation algorithm, which is based on a Hidden Markov Model approach for calling genetic event. Nexus standard configuration for gain and loss calling thresholds were used. All files have been reviewed for correct diploid calling according to the log2ratio and B-Allele frequency of each sample and have been adjusted if calling was not correct. Gains that include a minimum of 50 probes and losses that include a minimum of 25 probes were called. Furthermore, to remove false positive alterations, we performed filtering parameters provided by Nexus that includes normal structural variation listed in the Database of Genomic Variants (DGV, <http://dgv.tcag.ca> (5)) so that those calls, which have the same size and region listed as normal CNV, are automatically removed.

**RNA sequencing**

Data were processed and analyzed using the Mayo Clinic RNA sequencing in house analysis pipeline MAPRSeq (v2.1.1.) (6). Quality control was performed by using RSeQC (7). Briefly, 50-bp paired-end reads were aligned to human reference genome 37 by using Tophat (v2.1.0), and the counts per gene and per exon were summarized using HTSeq (8) using ENSEMBL gene v78. The Log2 transformed reads per million per kb (RPKM) were generated for gene differential expression analyses. For all the counts and RPKM, Ensemble (release-78) gene definition was used.

**Cell Line Authentication**

To ensure lack of cross contamination we have used a SNP panel identification assay to individually identify cell lines. All cell lines received into the laboratory are “fingerprinted” using a group of 33 single nucleotide polymorphic (SNP) variants and results are recorded in our cell line database. If cell line authenticity is questioned, the SNP assay is performed and results are compared. All contaminated cell lines are discarded.

**Antibodies**

Antibodies used in this study include anti-A20 Cell Signaling #5630, anti-Actin Santa Cruz sc-1616, anti-NF-κB Santa Cruz sc-372 (Santa Cruz, Santa Cruz, CA, USA), anti-MYD88 Cell Signaling #4283, anti-TRAF6 Abcam ab33915, anti-BCL2 Cell Signaling #2872, MYC Cell Signaling #9402, anti-p-p38 Cell Signaling #9211, anti-pNF-κB Cell Signaling #8242, anti-pSTAT3 Cell Signaling #9134, anti-STAT3 Cell Signaling #9139 (Cell Signaling, Danvers, MA, USA), and anti-p38 Millipore MABS1754 (Millipore Sigma, Darmstadt, Germany).

1. Lohr JG, Stojanov P, Lawrence MS, Auclair D, Chapuy B, Sougnez C, et al. Discovery and prioritization of somatic mutations in diffuse large B-cell lymphoma (DLBCL) by whole-exome sequencing. Proc Natl Acad Sci U S A. 2012;109(10):3879-84.

2. Ansell SM, Hodge LS, Secreto FJ, Manske M, Braggio E, Price-Troska T, et al. Activation of TAK1 by MYD88 L265P drives malignant B-cell Growth in non-Hodgkin lymphoma. Blood Cancer J. 2014;4:e183.

3. Li H. Aligning sequence reads, clone sequences and assembly contigs with BWA-MEM. arXiv:1303.3997v1 [q-bio.GN]. 2013.

4. Kocher JP, Quest DJ, Duffy P, Meiners MA, Moore RM, Rider D, et al. The Biological Reference Repository (BioR): a rapid and flexible system for genomics annotation. Bioinformatics (Oxford, England). 2014;30(13):1920-2.

5. MacDonald JR, Ziman R, Yuen RK, Feuk L, Scherer SW. The Database of Genomic Variants: a curated collection of structural variation in the human genome. Nucleic Acids Res. 2014;42(Database issue):D986-92.

6. Kalari KR, Nair AA, Bhavsar JD, O'Brien DR, Davila JI, Bockol MA, et al. MAP-RSeq: Mayo Analysis Pipeline for RNA sequencing. BMC bioinformatics. 2014;15:224.

7. Wang L, Wang S, Li W. RSeQC: quality control of RNA-seq experiments. Bioinformatics (Oxford, England). 2012;28(16):2184-5.

8. Anders S, Pyl PT, Huber W. HTSeq—a Python framework to work with high-throughput sequencing data. Bioinformatics (Oxford, England). 2015;31(2):166-9.
